# The Adult Neurogenesis Theory of Alzheimer’s Disease Predicts Where Tauopathy Develops in the Brain

**DOI:** 10.3390/ijms27198776

**Published:** 2026-09-30

**Authors:** Carlo Abbate

**Affiliations:** Fondazione Don Carlo Gnocchi ETS, Istituto Palazzolo, Via Don Luigi Palazzolo 21, 20149 Milan, Italy; cabbate@dongnocchi.it; Tel.: +39-02-39703657

**Keywords:** Alzheimer disease, adult neurogenesis, tau, neurofibrillary tangles, rehabilitation

## Abstract

Adult neurogenesis is impaired in Alzheimer’s disease (AD) and contributes to neuronal and cognitive dysfunction. Furthermore, efficient neurogenesis promotes cognitive resilience. I recently proposed a theory suggesting that neurogenesis plays a more central role in the pathogenesis of AD. The main hypothesis posits that new neurons harboring tau seeds propagate tauopathy as they mature and integrate into the brain. The aim of this study is to demonstrate that the theory successfully predicts the distribution of the earliest foci of tauopathy in AD. To this end, I synthesized data from the literature regarding the earliest localizations of tauopathy in AD. I then indicated where one would expect to find the earliest seeds of tauopathy if the theory were true. Finally, I compared the localization data with the theory’s predictions. Overall, the theory’s predictions were entirely consistent with the initial localization of tauopathy. Specifically, the theory is consistent with the exclusively neuronal and somatodendritic involvement of tauopathy; the preference for layer II and the transentorhinal/entorhinal complex; the early involvement of subcortical nuclei; phenotypic heterogeneity; differences between early- and late-onset AD; hemispheric asymmetries; and clinical-anatomical convergence. The theory demonstrates predictive power, economicity, compatibility with other theories, and generalizability to other diseases.

## 1. Introduction

A well-established finding in the study of the relationship between adult neurogenesis and Alzheimer’s disease (AD) is an early decline in neurogenesis as the disease develops. A reduction in neurogenesis was demonstrated in several studies using mouse models of AD [1], and was subsequently confirmed in human studies as well. In particular, an immunohistochemical study showed a loss of immature doublecortin-positive neurons [2], and a subsequent study using single-nucleus RNA sequencing (snRNA-Seq) confirmed impaired adult hippocampal neurogenesis (AHN) in patients with AD [3]. In a more recent study, the number of neuroblasts and immature neurons appeared reduced in a group of elderly individuals with AD compared to young adults, despite an increase in neural stem cells [4].

In light of the observed decline in adult neurogenesis in AD, a key question that has been raised in the field is whether this impairment is involved in the cognitive decline caused by the disease [5]. In this regard, a previous study using a mouse model of AD had already shown that blocking AHN exacerbated neuronal loss and cognitive decline, whereas inducing AHN in combination with brain-derived neurotrophic factor improved cognitive function despite a stable amyloid burden [6]. In a human study, some authors then found that a higher number of neuroblasts correlated with better overall cognitive scores in cohorts composed of subjects with both Mild Cognitive Impairment (MCI) and AD [7]. Consistent with this finding, some researchers found that a small group of individuals known as “resilient,” who remain cognitively intact despite their brains showing a significant burden of AD neuropathology, exhibited greater neurogenesis in the dentate gyrus (DG) of the hippocampus compared to patients with AD and MCI [8]. Furthermore, a more recent study suggested that immature cells actively contribute to the preservation of cognitive functions in the brains of resilient AD patients, as they were involved in transcriptional programs and intercellular interactions associated with protective signaling pathways (e.g., anti-inflammatory, neurotrophic, neuroprotective, myelinating, anti-apoptotic, and anti-amyloidogenic) [9]. Therefore, the consensus in the field is that impaired adult neurogenesis is a factor contributing to the neuronal and cognitive dysfunction caused by AD pathology. At the same time, data suggest that AHN may play a critical role in building cognitive resilience during the course of AD [5]. Thus, a second important question that has been raised in the field is whether enhancing adult neurogenesis (AHN) could serve as a treatment or rehabilitation option and improve cognitive dysfunction in AD [5,10,11].

In recent years, I have formulated a theory (i.e., the adult neurogenesis theory of AD) that posits that adult neurogenesis not only contributes to cognitive decline in AD once the disease is underway, but also plays a central role in the pathogenesis of AD at the onset of the disease [12]. In short, theory’s main hypothesis is that newly formed neuroblasts harboring tau seeds propagate tauopathy as they mature and integrate into the brain, through a “Trojan horse”-like mechanism, as some authors have referred to it [13,14]. In developing neurons within adult neurogenesis niches and/or in neurogenesis without cell division, all the necessary elements that predispose to pathological tau hyperphosphorylation and lead to the formation of tangles would in fact be present. Beyond the difficulties in validating the theory, which remains highly speculative, the aim of this work is to show how the hypothesis that the adult neurogenesis-migration system is closely involved in the tau pathogenesis in AD appears entirely consistent with, and/or succeeds in predicting, the multiple locations where tauopathy has actually been discovered in the early stages of the disease.

Studying where tauopathy occurs in the brain in AD is important because tauopathy is closely linked to neurodegeneration, as demonstrated by robust pathological evidence [15,16]. The distribution of neurofibrillary tangles (NFTs) in the cerebral cortex closely corresponds to the pattern of cerebral atrophy and hypometabolism indicative of cortical damage [17,18]. Furthermore, in vivo PET studies visualizing tau pathology have confirmed atypical distributions of NFTs, with increased tau PET signal values in brain areas (i.e., occipital and parietal cortex, left temporo-parietal region, perirolandic areas) corresponding to known phenotypic variants of AD (posterior cortical atrophy PCA syndrome, logopenic aphasia, corticobasal syndrome CBS, respectively) [17,18,19,20,21,22,23,24]. Given that tauopathy shows a fair degree of correspondence with the locations where neurodegenerative damage emerges in the brain in AD, studying where tauopathy emerges can help elucidate the causes of the distribution and nature of neurodegenerative targets. The reasons behind the anatomical localization of these targets of neurodegeneration remain important open questions in the field. Deciphering the anatomy of tauopathy in AD could therefore be key to understanding the ultimate pathogenesis of AD.

Given the exploratory nature of this work, I have allowed for a high degree of flexibility both in the different stages of tauopathy considered and in the micro- and macroscopic levels of the anatomical data. Thus, the data on the anatomical localization of tauopathy in the manuscript do not refer exclusively to NFTs but may also indiscriminately consider the preceding stages, from hyperphosphorylated tau monomers that self-aggregate to form dimers, followed by further aggregation into oligomers, pre-tangles, and NFTs [25]. At the same time, on the anatomical level, I considered the localization of tauopathy across different planes, ranging from different compartments within the cell, to different cell types, to cortical layers, to subcortical nuclei, to the medial temporal lobe (MTL), and finally to epicenters in specific cortical regions. Furthermore, I included some related issues such as differences in the anatomical targets of tauopathy in AD between adults and the elderly, hemispheric asymmetries, and the overlap of certain tauopathy targets in AD with other diseases.

In Section 2 of this article, I summarize the data on the main brain locations of pathological tau inclusions in AD, focusing primarily on the early stages of the disease. For each of these locations, I briefly outline some of the main hypotheses proposed in the field to explain them and/or the main challenges they face. A detailed review of the hypotheses proposed to explain each of the anatomical data related to tauopathy in AD, and an analysis of their plausibility, is beyond the scope of this work. In Section 3, I briefly outline the theory of adult neurogenesis. Finally, in Section 4, I describe how the new theory makes a series of predictions regarding the anatomical distribution of tauopathy in the brain that are entirely consistent with much of the known data on the localization of tauopathy in the early stages of AD.

## 2. First Localizations of Tauopathy in AD and Further Related Anatomical Issues

### 2.1. Tauopathy in the Somato-Dendritic Region

Under physiological conditions in adult neurons, the tau protein is primarily localized in axons, where it interacts with microtubules to maintain their stability and facilitate axonal transport [26,27] and to a significantly lesser extent in the soma and dendrites, including dendritic spines [28,29]. More specifically, studies in rat and bovine brains have shown that in the brain’s white matter—where axons predominate—the amount of tau is three times higher than in the gray matter, where cell bodies and dendrites predominate [30]. The distribution of tau in the axon under physiological conditions is not homogeneous but appears to follow a gradient, with the majority found in the distal part of the axon and lower concentrations in the proximal segment [31,32]. The localization of tau in AD differs from what is observed under physiological conditions in adults. Hyperphosphorylated tau, which precedes the formation of NFTs, accumulates primarily in the cell body and dendrites, with an initial preference for the distal portions of the dendrites [33,34]. According to the classical view in AD, hyperphosphorylated tau detaches from microtubules in the axon and then mislocalizes to the cell body and dendrites, where it ultimately forms neurofibrillary tangles [35,36].

### 2.2. Tauopathy in Different Cell Types

A second anatomical aspect of tauopathy in AD is its presence in different types of cells. Although pathological tau in AD is found primarily in neurons, it also appears in smaller quantities in glial cells, such as oligodendrocytes, astrocytes, and microglia [37,38]. However, the main source of tauopathy found in glial cells is external. Astrocytes, oligodendrocytes, and microglia can in fact internalize extracellular tau aggregates released by stressed or dying neurons, and in some cases, they fail to degrade tau, leading to secondary tau accumulation [39,40,41]. Unlike other tauopathies, in AD none of the major glial cell types—astrocytes, microglia, or oligodendrocytes—develop their own pretangles or tangles as a primary feature of the disease [42]. Other cell types, such as endothelial cells, are affected by the negative effects of tau from the outside [43] or may similarly internalize soluble tau aggregates but do not develop tangles [44].

### 2.3. Tauopathy in Layer II

The first cells to accumulate NFTs in AD are the stellate cells and large pyramidal projection neurons located in layer II of the trans-entorhinal cortex (TEC) [45]. Also, in the entorhinal cortex (EC), which is affected in the early stages of AD [46], there is a selective loss of excitatory neurons (ExN) expressing RAR-Related Orphan Receptor B (RORB) [47], and reelin [48] primarily in layer II, as well as in the superficial parts of layer III and in layer IV [49]. Interneurons (InN) in layer II of the EC and in the superficial part of layer III, particularly those expressing parvalbumin (PV), are also highly vulnerable [50]. In the neocortex, the associative areas are relatively vulnerable compared to other areas [46], and recent studies have established at the molecular level that the most vulnerable neurons in these areas are somatostatin (SST)-expressing interneurons [51,52,53,54], which are found with the highest density in layers II, III, V, and VI of the cerebral cortex [55,56,57], followed by layer II excitatory neurons expressing Cut Like Homeobox 2 (CUX2) and RORB [51,58]. However, RORB-expressing layer V neurons are also selectively vulnerable [51,58,59]. This brief review clearly shows that, beyond intrinsic molecular factors related to different types of vulnerable neurons, tauopathy has a particular tendency to target cells located in layer II, especially those in the medial temporal region, but also in the cortex.

One proposed explanation identifies neural connectivity as a key factor in this predisposition of tauopathy to target layer II [60]. Neurons in layer II contribute primarily to corticocortical connectivity, especially when combined with layer III neurons, thereby facilitating intracortical communication and the integration of information across cortical areas and hemispheres [61]. In particular, the EC layer II neurons form the origin of the perforant pathway, sending long axons to the dentate gyrus and cornu ammonis (CA) fields, facilitating trans-synaptic tau propagation from neocortical inputs [62,63]. High synaptic load and activity in this medial temporal hub amplify tau seeding in association cortex inputs, explaining initiation here before hippocampal spread [64].

### 2.4. Tauopathy in Transentorhinal/Entorhinal Cortex (TEC/EC) and Its Spreading in MTL Subregions

In classical amnestic AD, tauopathy begins in the medial temporal lobe [65]. More specifically, as previously reported, tau aggregates initially appear in stellate cells and large pyramidal neurons of layer II in the TEC (Braak NFT stage I) [45,46]. Then, through anterograde transmission from a presynaptic to a postsynaptic neuron along the axon, the neurofibrillary pathology spreads from the transentorhinal region into the olfactory bulb (OB) [66], the entorhinal cortex (EC), which has a major reciprocal connection with the TEC [67] and the hippocampal formation (Braak NFT stage II) [68,69,70]. As previously reported, the first cortical neurons to become involved in the EC are the multipolar neurons of the superficial layer II [71]. From the EC, via the same anterograde neural transmission pathway and through the connections of the perforant path, the involvement of two regions of the hippocampus—CA1 and the subiculum—is then described (Braak NFT stages II and III) [72,73]. Other regions connected to the EC via the perforant path, such as the DG and CA3 [72,73], are not affected early on but only later in the progression of the disease (Braak NFT stage III-IV) [74].

Both cell-autonomous and non-cell-autonomous factors have been identified as among the main causes of the vulnerability of regions of the middle temporal lobe to tauopathy [75]. With regard to cell-autonomous factors, it has been found that the first and most vulnerable neurons in AD are the excitatory neurons that express RORB and reelin in layer II of the EC [47,48]. Interneurons in layer II of the EC and the superficial portion of layer III are also vulnerable [50]. In particular, those expressing parvalbumin (PV) are more vulnerable than those expressing calbindin (CB), while those expressing calretinin (CR) are the least vulnerable [50,75]. One of the non-cell-autonomous factors considered relates to connectivity. Neurons in layer II, especially in the EC, contribute primarily to corticocortical connectivity [61]. The high synaptic load and activity in these hubs amplify the spread of tau protein, explaining why tauopathy initially develops in these regions before spreading further [64].

The classical view of the initial spread of tauopathy in the medial temporal regions—based on Braak staging [68,74] and the hypothesis of cell-to-cell transmission of tau [76,77]—is not without its limitations and inconsistencies.

First of all, we must remember that the amyloid theory—which is the primary and most widely accepted theory regarding the pathogenesis of the disease—lacks a clear explanation for why tauopathy begins in the TEC/EC complex. Following the amyloid cascade model, plaque deposition has a downstream effect on the formation of tangles; therefore, the area where the first tangles appear should correspond to an area of greater and preferential amyloid plaque deposition [78,79]. This prediction is, however, contradicted by the data. Beta-amyloid (Aβ) plaques, in fact, appear early in the associative cortex of the temporal lobe, which is located relatively far from the TEC where the first damaged neurons containing tangles appear [68,80]. Furthermore, studies have found that in the EC, which undergoes degeneration just as early as the TEC, beta-amyloid levels are no higher than those found in other, less affected brain regions [81,82].

A second limitation of the classical model concerns the spread of tauopathy between the TEC and the EC. The TEC has reciprocal connections with the entorhinal cortex (EC) [67]. In particular, the main output from the perirhinal cortex (PRC) [83,84] which includes the TEC terminates primarily in layer III of the EC with weaker innervation of the deep layer I at the border with layer II [85]. In contrast, input from the EC terminates in layer II and layers V–VI of the perirhinal cortex (PRC), which includes the TEC region [83,84]. Given this connectivity profile, the classical explanation for the anterograde transmission of tau pathology from the TEC to the EC would predict initial involvement mainly in layer III of the EC, where the output connections from the TEC to the EC terminate [83,84]. In contrast, the first tangles in EC appear in layer II [74,86,87].

A third issue in the classical model concerns the subsequent stage of tauopathy spread from the EC to the hippocampal regions. The main outgoing projection fibers from the EC constitute the perforant path and originate primarily from neurons located in layers II and III, although a minor component originates in layers V and VI [62,63]. These constitute the main input to the DG and an important input to the hippocampus and subiculum [62,63]. Furthermore, neurons in layers II and III of the EC project their axons in a rather selective manner. In layer II, stellate cells project to the DG and CA3, while the axons of layer III pyramidal cells target CA1 and the subiculum [72,73]. More specifically, cells in layer II of the EC project to the medial and lateral portions of the DG molecular layer [85], and the inputs target dentate granule cells [88] and interneurons in the molecular layer [89]. In turn, excitatory outputs from the DG target CA3, although neurons in layer II of the EC have collaterals that directly reach CA3, bypassing the DG [90,91]. Given this connectivity profile, the unresolved issue in the classical model concerns why, despite evidence that early tau pathology emerges in layer II cells of the EC at Braak stage II [74], and that these cells project to the DG and CA3 by the perforant path [62,63,72,73,85], the DG and CA3 are not affected by tau pathology at early stages. Indeed, granule cells of the DG remain unaffected at Braak stage III, and some tau pathology emerges only at stage IV [74]. Similarly, CA3, which receives afferents from the DG and directly from the EC [90,91], is not affected in the early stages of the disease [74]. Instead, tau pathology first emerges in CA3 at Braak stages III–IV [74]. This question is even more puzzling given that damage to the perforant pathway between the lateral EC and DG occurs unusually early in the disease process [92]. The long axons of projection neurons are, in fact, not well equipped to degrade or eliminate pathological protein aggregates [93].

A related aspect of this same unresolved issue is why tangles accumulate so early in CA1 and the subiculum (Braak stages II and III) and much earlier than in the DG and CA3 (Braak stages III and IV) [74] considering that receive afferent from perforant path fibres originating in entorhinal layer III of EC [72,73], which is not the first layer involved. Furthermore, the difference between regions affected early (subiculum and CA1) and those affected later (DG and CA3) cannot be explained by greater vulnerability of the different connecting fibers in the perforant path. In fact, it worth noting that entorhinal calbindin-positive neurons projecting to several targets including CA1 and the contralateral EC, the olfactory bulb and piriform cortex [94,95,96] are not found to be more vulnerable than reelin-positive neurons projecting to DG [48,97].

### 2.5. Tauopathy in Subcortical Nuclei

Another important anatomical feature of AD is early degeneration, including the presence of tau tangles, reduced neuronal viability, and cellular and synaptic loss, in the Neuromodulatory Subcortical Systems (NSS) [98]. Many studies have found that a large number of subcortical nuclei show early accumulation of tau tangles or pre-tangles, and in many cases even before involvement of the TEC/EC complex [99,100,101,102]. These nuclei include Meynert’s cholinergic basal nucleus (NbM), the noradrenergic locus coeruleus (LC), the dopaminergic ventral tegmental area (VTA), the orexinergic lateral hypothalamus (LH), and the serotonergic dorsal raphe nucleus (DRN). For example, the number of neurons in the LC containing hyperphosphorylated tau increases significantly when progressing from Braak stage 0 to stage I [103]. Moreover, the LC is the site of the earliest neurofibrillary pathology in over 20% of cases at Braak stage 0 and in all cases at Braak stage I [103]. Other NSSs accumulate hyperphosphorylated tau before the TEC [99,100,101,102], although for the DRN, this is estimated to occur slightly later than in the LC [103,104]. Over 20% of cases at Braak stage 0 and all cases at Braak stage I show neurofibrillary pathology in the DRN [104]. This nucleus is the earliest site of neurofibrillary pathology in 6% of all AD cases [104].

The reasons for the vulnerability of subcortical neurons to tau pathology are attributed primarily to their metabolic demands and to the oxidative stress resulting from neuromodulatory activity (e.g., noradrenergic activity in the LC), and long, unmyelinated axons prone to proximal misfolding [105,106,107].

It is important to note that the amyloid cascade hypothesis does not explain the early presence of tau pathology in the NSS. In fact, the amyloid pathology that, according to the model, should precede and drive tau pathology is instead absent in the early stages in these anatomical regions. Autopsy studies have indeed shown that amyloid plaques are found in the brainstem only in later stages of the disease [68]. However, there are exceptions, as some evidence shows early involvement of amyloid pathology with neuritic plaques in the striatum [108,109]. Nevertheless, some data suggest that synaptic damage in these areas is more closely associated with early accumulation of intracellular Aβ rather than with amyloid plaques [110].

### 2.6. Cortical Tauopathy and Phenotypic Heterogeneity

It is well known that tauopathy and neurodegeneration in AD can affect different cortical regions and, consequently, manifest as distinct clinical-cognitive phenotypes in different patients. Autopsy studies, atrophy studies, and more recent tau-PET imaging studies all point to four distinct patterns of tangle deposition and associated cortical damage in AD: hippocampal sparing, typical AD, limbic-predominant, and mild atrophy [17,111,112,113,114,115,116,117,118,119,120,121,122,123]. Furthermore, regarding the heterogeneity of cognitive phenotypes, the most common phenotype—especially in late onset AD (LOAD)—is the classic amnestic type. In addition to this phenotype, phenotypic variants are recognized that may be more frequent than the typical amnestic form in early onset AD (EOAD) [124,125]. In particular, there are variants known as PCA syndrome [126], logopenic [127], corticobasal syndrome [128] and behavioral/dysexecutive variant [129]. For some of these variants, tau-PET imaging studies have confirmed that the highest burden of tau pathology was in brain regions closely corresponding to clinical phenotypes. Specifically, in the posterior occipital-temporal and/or occipito-parietal regions for PCA syndrome [19], in the left temporo-parietal and/or perisylvian regions for the logopenic variant [20], in perirolandic regions for CBS-AD [21,22], and in the anterior regions for the frontal behavioral-executive variant [130,131]. The phenotypic heterogeneity of AD encompasses additional phenotypes, as demonstrated in a few but well-studied cases with semantic dementia syndrome (SD) (case 4 [132]), and non-fluent primary progressive aphasia (case PG [133]). Furthermore, in some studies by our group involving a small number of patients with biomarkers confirmed AD, we have suggested the emergence of additional possible phenotypes, including a right-hemispheric variant [134]. Finally, there are other less clearly defined phenotypes that should not be excluded from the study of phenotypic heterogeneity in AD, such as those corresponding to MCI subtypes (amnestic or non-amnestic multi-domain) [135]. It seems reasonable to expect, even for these rarer and as yet unconfirmed cognitive phenotypes of AD, that the distribution of tauopathy correlates closely with the localization of the epicenters of degeneration and consequent cerebral atrophy and/or hypometabolism. Therefore, the heterogeneity of tauopathy distribution in the cortex in AD may be greater than previously recognized.

Phenotypic heterogeneity in familial forms of AD (FAD) is lower than in sporadic AD, because genetic factors—ranging from the specific gene involved (Amyloid Precursor Protein APP, Presenilin 1 PSEN1, or Presenilin 2 PSEN2) to the type of mutation—likely help to standardize the phenotype within the same family, thereby partially reducing variability [136]. However, there is well-documented considerable inter-family variability among families with different mutations and also intra-family variability among carriers of the same mutation, both in terms of age of onset and clinical profile [137]. Furthermore, beyond differences in the degree of phenotypic heterogeneity, the brain regions affected by tauopathy and the resulting neurodegeneration in FADs and sporadic AD are entirely overlapping, although the age of symptom onset is much earlier in FADs [138]. In fact, FADs also present with the temporal-mesial (classical amnestic) phenotype, frontal (dysexecutive and behavioral) phenotypes, parietal (apraxia, dyscalculia) phenotypes, posterior occipital (e.g., PCA syndrome) phenotypes, and phenotypes involving the left-hemisphere (e.g., logopenic, other types of aphasia) [139,140]. Differences do emerge, however, in the relative frequency of phenotype onset among different mutations; thus, with APP gene mutations, the amnestic phenotype predominates at onset, whereas with PSEN1 mutations, non-amnestic phenotypes manifest more frequently at onset [139]. The main subcortical targets of tauopathy in FAD are also the same as those involved in sporadic AD (i.e., locus coeruleus, raphe nuclei, substantia nigra/ventral tegmental area, nucleus basalis of Meynert, thalamus, hypothalamus, subthalamus, striatum, and amygdala) [141]. The most significant differences between FAD and sporadic AD therefore do not concern so much the topography of tauopathy and the resulting neurodegeneration as they do the age of onset and the burden of pathology. In fact, FAD is characterized by a higher burden of amyloid and tau pathology [142], including more pronounced subcortical (striatal) involvement of Aβ pathology [143] and a higher frequency of associated CAA [144]. The clinical course appears more aggressive and rapidly progressive [145]. Furthermore, particularly with PSEN1 mutations, associated neurological signs emerge that are mostly absent in sporadic AD (e.g., myoclonus, epilepsy, parkinsonism, ataxia, spasticity, pyramidal signs) [140].

To date, no single explanation has been found for phenotypic heterogeneity in AD; rather, research highlights multiple contributing factors driving the phenomenon. Major drivers include genetic factors such as apolipoprotein epsilon-4 (APOE ε4) alleles, sex, age, and polygenic risk scores, which modulate symptom onset and progression [113,146,147]. Even identical genetic mutations, such as in presenilin 1 (PSEN1), produce variable phenotypes, suggesting epigenetic or environmental influences [148]. Recent studies emphasize tau pathology distribution and microglial heterogeneity as key modulators of neurodegeneration patterns and clinical variability [149,150]. Demographic factors and concomitant non-AD pathologies (e.g., vascular changes) further amplify this diversity [113].

It is important to note here that the amyloid cascade hypothesis [78,79] does not seem suitable for explaining the phenotypic heterogeneity in AD. As already reported, AD manifests with different clinical-radiological or anatomical syndromes, which encompass distinct cognitive phenotypes closely associated with patterns of cerebral hypometabolism and atrophy in the underlying brain regions [17,151,152,153]. However, this heterogeneity does not reflect a regional accentuation of Aβ deposition as the amyloid cascade would predict. Rather, the distribution of Aβ deposits in the brain is similar across all AD phenotypes, from autosomal dominant AD (ADAD) to cases of early onset AD (EOAD) and late onset AD (LOAD), with a single exception for Aβ deposits in the striatum in ADAD. In particular, the distribution of plaques invariably affects large, confluent areas of the associative cortex that overlap with a series of brain regions active at rest (default mode network) [154,155,156,157,158].

Similarly, the Braak staging [46,68,74,86] and the hypothesis of cell-to-cell transmission of tau protein [159] do not appear to adequately explain the phenotypic heterogeneity in AD. The model, in fact, predicts a single standard and stereotypical pathway of tangle deposition over time for all cases of the disease, which begins in the MTL and then spreads anterograde to various brain areas following the network of connections between neurons [46,68,74,86]. However, it is now known that the accumulation of tangles in phenotypic variants of AD primarily involves, and therefore likely originates in, various regions of the cortex rather than the MTL [17,19,20,21,22,111,112]. Furthermore, recent PET imaging studies of tau have confirmed that in some of these cases there is no involvement of the MTL [23,112,119,160,161].

### 2.7. Localization of Tauopathy in EOAD Versus LOAD

An important aspect of studying the anatomical distribution of tauopathy in the early stages of AD is that the sites affected by the disease vary depending on the patient’s age. In general, early onset Alzheimer’s disease (EOAD) is more likely to present with non-amnestic atypical clinical phenotypes compared to late onset Alzheimer’s disease (LOAD) patients [124,125,162,163,164]. Depending on the study, approximately 22–64% of EOAD cases are non-amnestic variant phenotypes [125,165,166,167,168,169,170,171,172] compared to 6% of those with LOAD [162,173]. Accordingly, EOAD primarily affects regions of the dorsal cortex, with 60–70% showing an atypical pattern of brain atrophy, whereas LOAD affects the MTL [124,170]. Given the close correlation found in tau PET imaging studies between clinical-anatomical syndrome and the localization of tau pathology in AD [17,111], the difference in clinical-anatomical syndromes between EOAD and LOAD reflect consistent differences in the sites of tauopathy deposition with age.

Current research has identified genetic factors, interactions between tau and amyloid-β (Aβ), and the properties of brain networks as main factors that determine the different localization of tauopathy in EOAD compared to LOAD.

Regarding genetic factors, first of all, some cases of EOAD (5–10% of cases) with very early symptom onset between the ages of 30 and 50 are carriers of autosomal dominant mutations in the amyloid precursor protein (APP), PSEN1, and PSEN2 genes, all of which contribute in different ways to increased amyloid pathology [174]. These cases show a higher burden of tauopathy with a frequent atypical fronto-parietal distribution, differing from the predominant limbic focus seen in LOAD [175]. Furthermore, the more numerous sporadic EOAD cases more frequently exhibit a genetic inheritance pattern containing rare variants in various endolysosomal (SORL1), immune (TREM2), and lipid-related (ABCA7, PLD3) genes, in addition to those associated with frontotemporal lobe degeneration (FTLD) (e.g., GRN, MAPT, C9orf72) that promote the spread of tau protein in the neocortex [24,175]. LOAD arises from sporadic Aβ accumulation and age-related factors, and multiple genes are involved as risk factors, including the well-known APOEε4, without deterministic mutations as in EOAD [175]. In such cases, tau is initially confined to the entorhinal/hippocampal regions [175]. In contrast, in EOAD, the frequency of the APOEε4 genotype is lower, and tauopathy shows a lesser tendency to affect the MTL, allowing hub-like patterns [24,175]. Considering Aβ-tau interactions, high Aβ burden in EOAD drives rapid, non-stereotypical tau aggregation via enhanced seeding and phosphorylation, promoting spread into association cortices [175,176]. In LOAD, weaker Aβ-tau synergy results in gradual, limbic-predominant tau that plateaus temporally due to co-pathologies (e.g., vascular, Lewy bodies) [175,176]. Finally, considering connectivity, some studies suggested that younger brains in EOAD exhibit higher vulnerability in connectivity hubs accelerating trans-synaptic tau propagation [177,178].

### 2.8. Hemispheric Asymmetries in Tauopathy

AD can present with asymmetric phenotypes in which tau pathology and neurodegeneration primarily affect a brain region in one of the two hemispheres, especially in the early stages, without involving—or involving to a lesser degree—the corresponding region in the other hemisphere. Autopsy studies and, above all, tau-PET imaging studies consistently reveal asymmetric tau pathology accumulation in both typical and atypical AD [17]. Moreover, this asymmetry tends to correlate with the dominant clinical symptoms (language vs. visuospatial), earlier onset, and more severe progression [179]. For example, considering aphasic variants of AD, it has been confirmed that the accumulation of tangles selectively affects certain regions of the left hemisphere in both the more common logopenic variant and other types of aphasia (e.g., agrammatic variant primary progressive aphasia PPA) [20,180,181]. Furthermore, although in cases of AD with a CBS phenotype the tauopathy burden often appears bilateral [21], some studies have shown clear asymmetries between the hemispheres in tauopathy burden [17,22]. Cases of AD with PCA syndrome also present with bilateral involvement of the posterior cortex; however, some studies have described patients with greater tauopathy deposition particularly in the right hemisphere compared to the left [180]. Even when considering the more typical forms of AD involving the temporal lobe, a recent large-scale study confirmed that tau pathology is often asymmetrically localized in the two cerebral hemispheres. Specifically, among 452 participants from the BioFINDER-2 cohort with tau- and Aβ-positive PET scans, 32% of patients with Alzheimer’s disease had tauopathy accumulation predominantly on the left (approximately 23%) or on the right (approximately 10%) [182].

The causes responsible for the asymmetric distribution of tauopathy in the cerebral hemispheres in some patients with AD have not been fully elucidated. However, in a recent study, some authors found that the degree of tau asymmetry was strongly associated with asymmetric amyloid-β (Aβ) patterns, not with differences in inter-hemispheric connectivity [182]. This finding suggests that regional vulnerability consistent with the amyloid cascade hypothesis, rather than network spread, drives asymmetry.

### 2.9. Tauopathy and Clinical-Anatomical Convergence

Another important aspect in the study of the localization of tauopathy in AD is clinical-anatomical convergence. AD, like other neurodegenerative diseases, exhibits this phenomenon, whereby the same anatomical region targeted by tauopathy in AD may also be affected by different diseases in other patients. Consequently, the same clinical cognitive phenotype may result from different underlying pathologies. For example, PCA syndrome may be primarily due to AD pathology, but also to Lewy body pathology, progressive supranuclear palsy (PSP), corticobasal degeneration, frontotemporal lobar degeneration-transactive response DNA binding protein 43 (FTLD-TDP-43) type A, Pick’s disease, and even a prion disease such as Creutzfeldt–Jakob disease [183,184]. CBS may be due to AD, corticobasal degeneration, Lewy body pathology, progressive supranuclear palsy, Pick’s disease, motor neuron disease (MND) dementia, nonspecific degenerative changes, and Creutzfeldt–Jakob disease [185,186].

The dominant account for clinical anatomical convergence in neurodegenerative diseases is that misfolded proteins spread along anatomically connected pathways, so that disparate starting pathologies progressively involve the same large-scale networks (e.g., default mode, salience, or frontoparietal networks) [187,188]. Certain network hubs (e.g., highly connected cortical regions, neuromodulatory centers like locus coeruleus or basal forebrain) are metabolically active and exposed to high levels of oxidative stress, making them selectively vulnerable across diseases [189]. When different proteinopathies hit these hubs, the outcome is similar: network dysfunction and a predictable clinical syndrome (e.g., frontotemporal dementia-like phenotype) [187,188].

### 2.10. First Localizations of Tauopathy in AD and Further Related Anatomical Issues: A Summary

In Table 1, I summarize all the initial sites of tauopathy in AD and further anatomical issues concerning the differences between EOAD and LOAD, hemispheric asymmetries, and clinical-anatomical convergence. Some of the main explanations proposed in the field to account for these sites are included. Figure 1 shows the first localizations of tauopathy in AD.

## 3. The Adult Neurogenesis Theory of AD

The main hypothesis in the adult neurogenesis theory of AD is that new formed neuroblasts harboring tau seeds propagate tauopathy during their maturation and integration in the brain [12]. In developing neurons within neurogenic niches and/or in neurogenesis without cell division (see Section 4.3), all the necessary elements are present that predispose to pathological hyperphosphorylation of tau and lead to the formation of tangles (Table 2) [12,190,191,192,193,194,195,196,197,198,199,200,201,202,203,204,205,206,207,208,209,210,211,212,213,214,215,216,217,218]. In the new theory, tau pathogenesis is relatively independent of Aβ pathogenesis; however, the latter plays an important indirect role in tau pathogenesis. The deposition of amyloid plaques, in fact, triggers the activation of glial cells, which in turn exert a significant pro-neurogenic effect by amplifying neurogenesis in the niches, reactivating immature cortical neurons, and supporting migration pathways that are largely quiescent in adults—analogous to what occurs in neurogenesis following other types of brain injury [12]. This strong pro-neurogenic action leads to a further increase in tau phosphorylation in immature neurons. This increase, coupled with a reduced clearance capacity in the neurogenic niche with aging and a hypothesized further increase in tau phosphorylation during migration in the inhibitory environment of the adult brain, underlies tau pathogenesis [12]. This scenario is also consistent with the amyloid cascade hypothesis, which posits a direct role for beta-amyloid in tau hyperphosphorylation. Recent data indeed show the presence of Aβ oligomers in immature neurons in the dentate gyrus in a mouse model of AD [219]. Aβ, tau, and even GSK-3β—the primary kinase mediating Aβ-induced tau hyperphosphorylation in AD—may therefore all be present in immature neurons during adult neurogenesis. The theory aims to explain the very earliest stages of disease onset, during which the first seeds of tau pathology develop and the epicenters of future neurodegeneration are selected. In this phase, the theory predicts that the positive, pro-neurogenic effects of glial cell activation predominate over the detrimental ones [220], and that tauopathy has not yet produced significant negative effects on adult neurogenesis itself [221].

## 4. The Adult Neurogenesis Theory of AD Is Consistent with Much of the Data on the Early Anatomical Distribution of Tauopathy

### 4.1. Somato-Dendritic Localization of Tau Protein in Immature Neurons

The hypothesis that the pathogenesis of tau occurs in developing neurons within neurogenic niches and/or in immature cortical neurons in adults is consistent with the initial localization of tauopathy in the distal parts of dendrites and the soma in AD, despite the fact that the tau protein is primarily localized in the axons of mature adult neurons.

First, while the tau protein is preferentially localized in axons rather than dendrites in mature neurons in healthy adults [26,27], at the time when neurons become polarized, it is distributed indiscriminately in both the axon and the minor processes [222,223]. Thus, in immature neurons, tau is indeed present in the dendrites and soma.

A second element supporting the new scenario is the role of the serine/threonine kinase 3 beta (GSK-3β). GSK-3β is a key player in the amyloid cascade; in vitro [224], and in vivo [225] studies have in fact demonstrated that Aβ exerts its harmful action by activating GSK-3β [204,205,224]. Consistent with this, increased GSK-3β activity has been observed in the brains of patients with AD [226]. These data confirm that GSK-3β is a key element in the pathogenesis of AD and support the idea that this kinase represents a crucial molecular link between Aβ and tau [204,205,227,228]. Of note here is that GSK-3β plays key roles in multiple aspects of neuronal development, including axonal growth and maintenance, neurogenesis, neuroprotection, neuronal migration, and neuronal polarization [229,230,231]. Even more important for the discussion here is the fact that in in vitro studies at an early stage of neuronal morphogenesis (stage 2), when the neuron is still multipolar, GSK-3β is localized at the tips of all neurites as well as the axon [231].

The third element that remains to be explained in the new model is the absence of tauopathy at the axonal level. In the classical view of AD pathogenesis, the presence of tauopathy at the dendritic level rather than the axonal level is explained by the fact that axonal tau from mature neurons detaches from microtubules and mislocalizes into the somatodendritic compartment [35,36,232]. In the new model, however, tauopathy is found at the dendritic level because it begins to develop when neurons are immature and still developing. At this stage of neuronal development, as noted above, tau and another key player such as GSK-3β are in fact present at the dendritic level. At this point, compared to the classical view, the new scenario lacks an explanation for why we do not find tauopathy at the axonal level in AD. My tentative explanation in this regard is that tauopathy in AD could begin in immature neural cells—whether quiescent in the cortex or in niches of adult neurogenesis—during a developmental phase preceding axon formation. The presence of hyperphosphorylated tau in the neuron’s processes then conflicts with the dynamics of axon specification, so that the axon, when it emerges, is free of tauopathy.

More specifically, it has been demonstrated both in vitro and in vivo that in the morphogenesis of developing neurons, axon specification is merely an intermediate event, preceded by phases in which neurons are multipolar, possessing a soma and multiple indistinct minor projections called neurites [233]. Among these neurites, one will then be specified as an axon, while the remaining ones will give rise to dendrites (in vitro) or a single leading process that will become a dendrite in the bipolar neuron (in vivo), completing the process of neuronal polarization [233,234,235,236,237]. Even so-called immature cortical neurons (see Section 4.3) are in a stage of maturation that precedes the development of an axon [238,239,240,241]. In fact, they include neurons of different morphologies, including type I cells with a small soma and a single short process, type II cells with a larger, multipolar soma, featuring one or two longer dendrites and a thin axon-like process, and finally neurons with intermediate characteristics [239,241]. However, none of these immature cortical cells, including type II cells with a thin axon-like process, express a marker (Ank-G protein) of the axonal initiation segment (AIS) [239], which is a structure related to neuronal maturation and identifies the axon [242,243].

Regarding the subsequent phase of axon specification, it is important to note first of all that although microtubules (MT) stability is not limited to the axon but also occurs in neurites, the future axon nevertheless exhibits more stable microtubules in the cell body with greater resistance to depolymerization compared to neurites [244]. Furthermore, microtubule stabilization is a critical event that plays an active role in inducing axon formation during early neuronal development [244,245]. GSK-3β is one of the kinases involved in regulating microtubule stability. In fact, GSK-3β regulates the affinity of microtubule-associated proteins (MAPs) for MTs through phosphorylation [246], which in turn control MT dynamics. For example, when GSK-3β is inhibited, the phosphorylation that inactivates MAPs decreases; as a result, MAPs bind more strongly to microtubules, which consequently become more stable [247,248]. Thus, in the developing neuron, axon specification among the neurites will occur in the one where MT stability is greatest. Confirming this fact, experimental inhibition of GSK-3β, which leads to MT stabilization, promotes the formation of multiple axons [249,250].

Since tau hyperphosphorylation contributes to decreased MT stability, and conversely, MT stability is a factor in neuronal polarization, consequently, in our scenario of initial tauopathy in the neurites of the developing neuron, axon determination/specification will occur in the neurite with the lowest load of pathological tau and/or greater GSK-3β inactivation. In other words, the neurite showing less tau hyperphosphorylation and/or where GSK-3β inhibition prevails will have more stable MT dynamics compared to the others and is therefore the one that will become the axon.

In summary, in the immature neuron, tau protein and GSK-3β are present at the somato-dendritic level. Furthermore, the presence of early pathological tau conflicts with the dynamics of axon specification. Thus, when the axon develops, it is free of pathological tau compared to the somatodendritic compartment.

At this point, it is important to note that some authors found clear evidence of intracellular Aβ oligomers generated in NSCs in the subgranular zone (SGZ) at a very early stage in a transgenic AD mouse model [219]. Thus, three key elements in the pathogenic model of AD predicted by the amyloid cascade—Aβ, GSK-3β, and tau protein—may all be present at the dendritic level in immature neurons. The scenario of early dendritic localization of tauopathy predicted by the theory of adult neurogenesis in AD is consistent with recent data highlighting the toxic role of tau protein at the dendritic level, with synaptic loss playing a more significant role in the pathogenesis of AD than the formation of tangles [251,252]. Furthermore, the results of some recent studies seem to perfectly complement this scenario by demonstrating a possible mechanism that contributes to the development and accumulation of tauopathy at the dendritic level in AD. In particular, some authors, using a new imaging technique (STARFISH) they developed to localize the site of endogenous tau protein synthesis with high spatial resolution, have shown in primary neurons from the mouse brain that the translation of endogenous tau occurs exclusively in the dendrites, near the synapses, and not in the cell body [253]. Furthermore, they found that proteasomes localized to the plasma membrane (neuroproteasomes) directly regulate the turnover of newly translated tau in dendrites. Selective inhibition of neuroproteasome function rapidly leads to the accumulation of tau and, surprisingly, to the de novo formation of sarkosyl-insoluble endogenous tau paired-helical-filaments (PHFs) with biochemical and ultrastructural characteristics similar to those of human PHFs in AD. A related study using the same basic approach then showed that selective inhibition of neuroproteasomes induces the formation of tau PHFs much more markedly under conditions that mimic the APOE4 genotype and advanced age [254].

### 4.2. Different Cell Types in Adult Neurogenesis and Tauopathy

In niches of adult neurogenesis, neurons are the primary cell type to develop, but glial cells such as oligodendrocytes and astrocytes also develop in smaller numbers [255]. In contrast, when quiescent immature cortical neurons (see Section 4.3) are activated, they develop exclusively into neuronal cells and not into other cell types [238]. Microglia and endothelial cells, on the other hand, have a different origin than oligodendrocytes and astrocytes and do not develop through adult neurogenesis [256,257].

Although adult neurogenesis in niches involves relative cellular heterogeneity that includes astrocytes and oligodendrocytes in addition to neurons, the new theory is entirely consistent with the fact that endogenous tauopathy is present exclusively in neurons in AD. In an adult brain, tau protein is indeed clearly expressed in mature, myelinating oligodendrocytes; however, immature oligodendrocytes such as oligodendrocyte precursor cells (OPCs) and pre-oligodendrocytes present in neurogenic niches generally do not express tau protein [258,259]. Accordingly, the presence of tau protein in oligodendrocytes is considered a marker of cell maturity [258].

Unlike mature oligodendrocytes, under physiological conditions endogenous tau protein is almost undetectable in astrocytes of normal brain tissue [260]. However, a minimal amount of Microtubule-Associated Protein Tau (MAPT) mRNA encoding the tau protein is expressed in astrocytes, and this amount may increase under pathological conditions of astrocyte activation, such as in AD [261]. Therefore, even though most pathological tau inclusions in astrocytes in AD are of external origin, endogenous tau inclusions can theoretically form. In adults, oligodendrocytes in particular, but also astrocytes, have the potential to form endogenous tau inclusions, as demonstrated in other tauopathies. For example, oligodendrocyte-specific tau pathology, in the form of fibrils and coiled coils, is evident in major tauopathies including progressive supranuclear palsy (PSP), corticobasal degeneration (CBD), and Pick’s disease (PiD) [262]. Similarly, in other tauopathies (e.g., progressive supranuclear palsy, corticobasal degeneration, argyrophilic grain disease, ARTAG), tau-positive astrocytic inclusions (e.g., tufted astrocytes, astrocytic plaques) have been documented even in areas where tau-related neuronal pathology is minimal, suggesting that at least a portion of this tau protein originates from the astrocytes themselves rather than solely from uptake [263,264].

According to the adult neurogenesis theory of AD, although oligodendrocytes and astrocytes have the potential to form endogenous tau inclusions, as occurs in other tauopathies, this does not occur in AD because these cells do not express the tau protein when they are immature.

### 4.3. Adult Neurogenesis Without Cell Division and Layer II

It is important to note that in adults, new neurons are not generated solely in neurogenic niches. In fact, a second and likely more efficient type of adult neurogenesis has been discovered, particularly in primates, known as neurogenesis without cell division [238,239,240,241].

In particular, neurons that express markers typical of immaturity and do not divide at all have long been discovered in adults of various mammalian species, including humans [238,239,240,241,265,266,267]. These cortical immature neurons (cINs) are generated before birth but delay their maturation and activation until adulthood, remaining dormant, in a standby state, in a state of prolonged immaturity [238,239,240,241,265,266,267]. However, they retain the potential to grow and differentiate into mature neurons—developing dendrites, spines, and synaptic inputs—and integrate into pre-existing neural circuits during adulthood, remaining stationary in their original location [238,239,240,241,265,266,267]. While in rodents with smaller, less gyrated brains, immature cortical neurons have been found only in the piriform and entorhinal regions of the paleocortex, in many mammals with larger, more gyrated brains—including humans—they have been found in multiple regions of the neocortex as well as in subcortical regions [238,239,240,241,265,266,267]. In particular, in humans from adolescence through adulthood, immature cortical neurons have been discovered throughout the entire cortex, in the frontal, parietal, and occipital lobes, and extensively in the temporal lobe—including the entorhinal cortex and the parahippocampal gyrus—as well as in subcortical regions, notably the amygdala [267]. In older adults, however, they persist primarily in the temporal cortex, at the entorhinal level, and in the amygdala [267].

The most significant finding for the theory of adult neurogenesis in AD proposed here is the fact that immature cortical neurons have been consistently and invariably localized across studies, scattered within layer II of the cortex [238,239,240,241,265,266,267], precisely where we know there is a predilection for the appearance of the first seeds of tauopathy in AD. The reason why these immature neurons are restricted to layer II is unknown; however, it is known that the superficial layers (II and III) of the cortex play an important role in providing cortico-cortical association projections [238,241]. It is therefore hypothesized that the presence of such neurons supports cortico-cortical connectivity, which is related to the greater importance in higher mammals of information integration between cortical regions and associative learning [238,241]. Accordingly, the superficial layers occupy a much larger portion of the cortex in primates and less space in rodents [268].

### 4.4. Adult Hippocampal Neurogenesis and Tauopathy in Medial Temporal Lobe

The strongest correlation between the theory of adult neurogenesis in AD and the anatomical distribution of tauopathy at disease onset lies in the fact that the location of the primary niche for adult neurogenesis coincides with the main site where tauopathy initially develops in most AD patients, namely the hippocampal regions. The primary niche for adult neurogenesis is, in fact, located in the subgranular zone (SGZ) of the dentate gyrus (DG) in the hippocampus [269]. Specifically, according to the new theory, the first seeds of tauopathy would develop in maturing neurons within the DG neurogenesis niche [12]. These tauopathy seeds would then be carried retrograde through the perforant path connections toward the EC and then, via the multiple connections between the two areas, from the EC to the TEC [12]. From that point on, the spread of the first tauopathy seeds follows Braak’s classic pattern of progression from the EC to the cortical regions. This new model of the development and initial spread of tauopathy in the medial temporal regions is consistent with extensive anatomical data and appears to overcome some of the limitations and inconsistencies left by the classical explanatory model.

First, in the theory of adult neurogenesis in AD, the hippocampal localization of the main neurogenic niche explains why tauopathy emerges precisely in these regions. Furthermore, although the first seeds of tauopathy emerge in immature neurons within the DG neurogenesis niche, tangles do not accumulate there early on due to the powerful clearance mechanism typically present in neurogenesis niches, which eliminates many of the new neurons containing the forming tangles. In particular, most newborn neural progenitors undergo apoptosis within a few days of being generated in the SGZ, and microglia phagocytose the apoptotic debris of these cells to help maintain the balance of the neurogenic niche [270]. In close correlation with the suggested inhibition of tauopathy in the DG, the formation of tangles would also be delayed in CA3, which receives major input from the DG [90,91]. In the new theory, the initial seeds of tauopathy from the DG would then be transmitted retrograde to the GABAergic interneurons of the molecular layer. It is well known, in fact, that adult-generated neurons in the SGZ of the DG receive local connections from various types of GABAergic interneurons [221], whose inputs to the niche are essential for maintaining a healthy level of neurogenesis under normal conditions [271,272]. It is important to note that these GABAergic interneurons have been shown to be particularly vulnerable to the formation of tau tangles in AD [221,273,274,275,276]. Furthermore, some authors, using both samples from AD patients and a transgenic mouse model (3xTg), have not only confirmed that pathological tau accumulates primarily in GABAergic interneurons [221], but also that the overexpression of hyperphosphorylated tau in DG excitatory neurons or in newly formed immature granule cells has minimal detrimental effects on neurogenesis in the adult hippocampus, supporting the idea that tau pathology in AD would be suppressed or at least delayed in certain DG cells. The retrograde transmission hypothesized to support the new model is recognized as to be possible. In fact, projection neurons generate a long axon to transmit information from one site to another, and to this end, their axons possess mechanisms for the anterograde and retrograde transport of various cargoes [93,277]. Furthermore, the idea of retrograde transmission of tau pathology in the MTL in AD has already been suggested by other authors [278]. From the DG, in a retrograde manner, the first seeds of tauopathy travel along the connecting fibers of the perforant path that link the DG with the EC, terminating precisely in layer II of the EC, where the first signs of tauopathy in AD are known to appear. Moreover, this scenario is consistent with the fact that the fibers of the perforant path between the lateral EC and DG undergo very early deterioration [92]. From the EC, through the multiple connections between the two areas, tauopathy would then be transmitted to the TEC in two ways: both anterograde and retrograde. In this way, a massive reception and accumulation of tauopathy seeds in the TEC would explain the early appearance of tangles in this region. Not only that, but this view is consistent with the fact that the first tangles in the TEC appear in layer II, which is the main destination of output fibers from the EC [83,84]. Furthermore, this diffusion model also explains the involvement of CA1 and the subiculum. In fact, as already noted in the new scenario, the fibers in the perforant path connecting the EC with the DG undergo early deterioration due to the passage of the first tauopathy seeds in a retrograde manner from the DG to the EC during the initial phase of spread. In subsequent phases, after the tauopathy seeds have affected the EC and TEC, they are then transmitted anterograde along the multiple output connections from the EC toward other regions, including the hippocampus via the perforant path. At this point, however, the scenario predicts that the only undamaged fibers available in the perforant path are precisely those toward the subiculum and CA1, since those between the EC and DG have already been damaged by the previous retrograde spread of tauopathy from the DG to the EC. Finally, to conclude the discussion on the medial temporal regions, it should be noted that the olfactory bulb (OB), which is affected early by tauopathy in AD [66,279,280,281], is one of the main sites of adult neurogenesis, as it is the endpoint of the canonical migration pathway of RMS cells originating from the SVZ niche [269].

### 4.5. Adult Neurogenesis Niches in Non-Canonical Locations and Tauopathy in NSS

The new theory of adult neurogenesis in AD also offers a possible explanation for the early presence of tauopathy in NSS, given that adult neurogenesis niches have been discovered in non-canonical locations in various mammalian species, including several subcortical sites [282]. For example, neurogenesis has been documented in the hypothalamus of various species of adult mammals, including rodents, mice, rats, and voles [283,284,285,286,287,288,289,290,291,292]. Furthermore, there is evidence of neurogenic niches in mammals also in the substantia nigra and the amygdala [282]. The lateral hypothalamus is one of the regions where tauopathy appears early in AD [99,100,101,102]. Furthermore, it must be noted that the hypothalamus is highly connected to the LC, which is another of the subcortical nuclei where tauopathy appears early in AD [103]. Other important subcortical nuclei that show early tauopathy in AD, such as the ventral tegmental area (VTA) and the nucleus basalis of Meynert (NBM), have multiple connections, including with the amygdala in the limbic system for both input and output and also with the hypothalamus for input [99,100,101,102]. Another nucleus involved early in tauopathy, such as the dorsal raphe nucleus (DRN) [103,104], also has connections with the amygdala. To complete the picture of the subcortical regions involved in adult neurogenesis, it is worth noting some data showing the presence of neurogenesis in humans within the striatum [293]. However, this region is not affected by tauopathy in early-stage AD [68]. Rather, it should be noted that the striatum may also exhibit early accumulation of intracellular Aβ in AD [108,109,110] and, in particular, shows a marked tendency for the accumulation of Aβ deposits in autosomal dominant AD (ADAD) [294]. In the context of the new theory, this finding raises the question of whether adult neurogenesis in this region is in any way related to amyloid pathology.

Even when considering neurogenesis without cell division, the available data support the presence of immature neurons in subcortical areas [240,266]. In particular, certain areas of gray matter such as the amygdala and the claustrum, and of white matter such as the external capsule and the corpus callosum, are rich in immature neurons, albeit with differences across species. For example, a high number of immature neurons has been found in the amygdala, particularly in non-human primates and in humans, whereas it appears modest or absent in mice [240,266].

### 4.6. Cortical Neurogenesis and Tauopathy (Phenotypic Heterogeneity)

In the theory of adult neurogenesis in AD, the presence of different neurogenic niches and/or the different locations of quiescent immature neurons provides a framework that seems well-suited to explaining the phenotypic heterogeneity of AD. First, the location of the main adult neurogenesis niche in the DG of the hippocampus accounts for amnestic phenotypes. Second, it is important to emphasize that, especially in the case of brain injury, immature neurons originating in the neurogenic niche of the SVZ can potentially reach every region of the cortex—occipital, frontal, temporal, and parietal—by following various long migration pathways [216,295,296]. Similarly, regarding cortical immature neurons, the data support their varied localization across different cortical regions, including the frontal, parietal, and occipital lobes, and extensively in the temporal lobe [267]. Thus, the neurogenesis-migration model appears suitable for explaining the emergence of the various clinical-radiological syndromes of AD, ranging from limbic-predominant AD with an epicenter in the hippocampus (classic hippocampal amnesic syndrome) to variants with epicenters in the posterior cortex (PCA syndrome), anterior cortex (frontal-dysexecutive and behavioral variants), parietal (CBS syndrome). The same model, hypothesizing an associated and simultaneous involvement of different niches with different migration pathways, or of different pools of immature neurons, is well-suited to explain the most common cases encountered in clinical practice, in which neurodegeneration—and thus likely the underlying tauopathy as well—probably begins in two or more distinct epicenters at the same time. These include phenotypes ranging from typical AD, in which the hippocampal epicenter is associated with a cortical epicenter resulting in cognitive impairment distinct from amnesia—as in cases of multi-domain amnestic MCI—to hippocampal sparing, where cortical targets predominate without hippocampal involvement—as in cases of multi-domain non-amnestic MCI. More generally, the presence of distinct neurogenic niches and/or pools of quiescent immature neurons is consistent with the possible simultaneous onset of the disease at different epicenters.

Given that the topography of cerebral targets—both cortical and subcortical—in FAD is virtually indistinguishable from that in sporadic AD [138,139,140], the neurogenesis-migration model also seems well-suited to explaining the distribution of tauopathy in familial cases of the disease. However, due to the massive burden of amyloid and tau pathology [142] and the extremely young age of disease onset [139], FAD—though rare—represents an important test case for the new theory, which requires further in-depth study in the future. Upon preliminary examination, in the face of a greater amyloid burden, the new model predicts a scenario of a more significant glial response, resulting in a more robust pro-neurogenic effect, and in turn, increased tau phosphorylation in immature neurons—which, in some ways, appears consistent with the observation of a greater tauopathy burden in FAD cases. However, early-onset age appears to pose a challenge for the theory, given that in such young patients we would expect more efficient neurogenesis with preserved clearance capacity in the niches and greater ease of migration compared to later-onset cases of sporadic AD. In this case, it is less clear why the pro-neurogenic action of glial activation should ultimately lead to a shift toward pathological forms of tau aggregation and deposition. However, one possible explanation might come from analyzing the important role that APP, PSEN1 and PSEN2 play in adult neurogenesis and, conversely, the negative impact that mutations in these genes have on neurogenesis in FAD. In particular, APP, through its potentiating effect on GABAergic tone, preserves an environment favorable to adult neurogenesis, promoting the proliferation, survival, and proper integration of new neurons [297], while PSEN1 is crucial for the maintenance of NSCs in niches (SGZ and SVZ) [298]. PSEN2 also contributes to the regulation of NSCs, although its role is less dominant than that of PSEN1 [299]. In contrast, APP mutations in FAD increase the production of Aβ42 and more aggregative forms of Aβ, which have toxic effects on progenitor cells and the neurogenesis niche [300]. PSEN1 mutations reduce Notch signaling in neural progenitors, promoting premature neuronal differentiation and a more rapid depletion of the NSC pool [298]. Therefore, the neurogenesis-migration model could also be adapted to explain cases of FAD with early-onset age by taking into account the negative effects that genetic mutations in APP, PSEN1, and PSEN2 have on adult neurogenesis. Figure 2 shows the main sites of adult neurogenesis.

### 4.7. Decreased Neurogenesis with Aging and Differences in the Localization of Tauopathy Between EOAD and LOAD

The difference in the primary brain localizations of tauopathy and the resulting neurodegeneration between EOAD and LOAD is explained in the new theory simply by the well-documented decline in the functionality of the neurogenesis-migration system during aging [301,302,303]. The age-related decline in the functionality of the neurogenesis-migration system involves the inactivation of the V-SVZ niche as early as adulthood, followed by a progressive decline in functionality with aging in the SGZ of the DG, which remains the only area of residual neurogenic activity in old age [302]. In particular, the new theory predicts that in some adults or young elderly individuals, the neurogenesis niche in the V-SVZ may still be active, or be promptly reactivated, in addition to that in the DG SGZ. In such cases, some of the neurons forming in the V-SVZ, by managing to undertake long migrations toward the cortex, carry the seeds of tauopathy to the different frontal, parietal, temporal, and occipital regions, developing epicenters of degeneration in the cortex (EOAD). Conversely, in the elderly, with only the niche in the DG SGZ remaining predominantly active, tauopathy and the resulting neurodegeneration would develop primarily at the hippocampal level (LOAD). The same scenario is confirmed when considering neurogenesis without cell division. For example, a neuropathological study documented the presence of immature cortical neurons in multiple sections of the frontal, parietal, and occipital lobes, and more consistently in the temporal lobe, in the brains of some donors aged 22 to 56 years [267]. In contrast, in the group of elderly donors aged 68 to 101 years, immature cortical neurons persisted only in the temporal cortex and amygdala [267]. Accordingly, the reactivation of quiescent immature cortical neurons carrying the first seeds of tau pathology may involve certain regions of the cortex rather than the hippocampal region in some adults or young elderly individuals, whereas it is less likely to occur among the elderly.

### 4.8. The Two Homologous Systems of Neurogenesis in the Cerebral Hemispheres and Asymmetries in the Localization of Tauopathy

The occurrence of asymmetries in the localization of tauopathy and/or the resulting degeneration between homologous regions of the cerebral hemispheres or between the two hemispheres as a whole in AD is consistent with the theory of adult neurogenesis in AD. Each cerebral hemisphere, in fact, has its own niches of neurogenesis, its own migration pathways, and its own populations of quiescent immature cortical neurons. There are two homologous systems of adult neurogenesis, each in its own hemisphere. Therefore, at least in theory, it is possible to conceive that these two systems may have a certain degree of independence and exhibit differences in function or efficiency. In that case, any cause that differentially interferes with the efficiency of one of the two adult neurogenesis systems compared to its homologue in the other hemisphere (e.g., lower or higher NSC production, less or more efficient autophagy/clearance in the niche, more difficult or easier migration, a smaller or larger pool of quiescent neurons, etc.)—all other conditions being equal—would directly result in a difference in tauopathy and/or consequent degeneration in the brain area of that hemisphere compared to its counterpart in the other hemisphere.

Although no hemispheric asymmetries in neurogenesis have been reported to date in mammals, including humans [304], clear asymmetries have instead been discovered in some songbirds [305]. For example, some authors have found in certain songbirds a much higher number of new neurons in a region of the forebrain (caudomedial nidopallium, NCM) involved in the discrimination and memory of conspecific vocalizations in the left hemisphere compared to the right [305].

Furthermore, differences in neurogenesis function between the two hemispheres have been clearly demonstrated in neurogenesis studies using animal models of various types of brain injury. For example, in one study, researchers experimentally induced lesions in the dopaminergic neurons of the substantia nigra (6-hydroxydopamine lesion model of Parkinson’s disease) in adult rats to simulate damage similar to that seen in Parkinson’s disease and administered infusions of transforming growth factor alpha (TGF-α) into forebrain structures [306]. In the injured animals that received a TGF-α infusion, rapid proliferation of stem cells was observed in the SVZ ipsilateral to the lesion, followed by migration of neuronal and glial progenitors toward the region of the TGF-α infusion site, and subsequently, the appearance of an increasing number of differentiated neurons in the ipsilateral striatum [306]. Similar hemispheric differences in neurogenesis and migration have also been demonstrated in adult rats with experimental models of ischemic stroke involving striatal damage induced by temporary occlusion of the middle cerebral artery [307,308]. In these studies, an increase in neurogenesis in the ipsilateral SVZ and migration toward the ischemic striatum were observed following the lesion.

Finally, the idea that the neurogenesis-migration system may function differentially between the two hemispheres is also indirectly suggested by data on brain developmental malformations in humans during childhood. In some cases, the neurogenesis-migration system malfunctions during development, affecting the proper arealization of the brain and resulting in known malformations. Such malformations sometimes affect only one hemisphere (e.g., left or right hemimegalencephaly, left or right unilateral polymicrogyria, etc.) [309] or appear most severe in a circumscribed region of only one of the two cerebral hemispheres, while the other hemisphere or the homologous area of the other hemisphere develops normally (e.g., left or right frontal polymicrogyria, left or right peri-sylvian polymicrogyria, etc.) [310].

### 4.9. Adult Neurogenesis, Clinical-Anatomical Convergence, and Tauopathy

According to the theory of adult neurogenesis of AD, tauopathy begins in maturing neurons. Consequently, the initial localization of tauopathy—with subsequent foci of degeneration in various areas and regions of the brain—depends on the location of adult neurogenesis niches and/or the directions taken by neuronal migration, or on the cortical localization of pools of immature neurons that are reactivated. The reactivation of the neurogenesis-related arealization genetic program could be the vector that provides the spatial instructions, or topographic markers, allowing for the selection of which area the pool of immature neurons is reactivated in, or toward which cortical region the first neuron(s) carrying the seeds of tau pathology are guided during migration from the adult neurogenesis niches [12]. Thus, in the new theory, the localization of tauopathy in AD depends on a system that generates the new neurons in which tauopathy develops and/or on instructions that guide them (like Trojan horses) toward different brain areas. The system that determines where tauopathy develops in the brain is therefore relatively independent of the pathogenic mechanism that causes tauopathy. This relative independence allows us to hypothesize that the same system may be involved in guiding the anatomical distribution in the brain for other proteinopathies as well. To give an example, any intraneuronal proteinopathy that develops in maturing neurons within the adult neurogenesis niche of the V-SVZ, which then migrate toward the occipital cortex, or similarly within the pool of immature cortical neurons already present in the posterior cortex which are reactivated, could explain the presence of a focus of posterior degeneration correlated with that specific proteinopathy. In such a case, we would have the basis for explaining the clinical-anatomical convergence of PCA syndrome.

The idea of a relative independence between the genesis of a proteinopathy and the system that determines its distribution in the brain opens the door to the hypothesis that other degenerative dementias may also share the same neurogenesis-migration system in their genesis. In this regard, it is intriguing to recall, considering Parkinson’s disease dementia (PDD) and limbic-predominant age-related TDP-43 encephalopathy (LATE), the discovery in some mammals of niches of neurogenesis in the substantia nigra and amygdala, respectively, which are the epicenters of degeneration in the two diseases [282]. Finally, the study of chronic traumatic encephalopathy (CTE) provides unexpected evidence that the neurogenesis-migration system may drive the topographic distribution of tauopathy in the brain in other diseases as well. It is important to note that, despite the difference in the pathogenesis of CTE and AD and the fact that phosphorylated tau fibrils in CTE exhibit a distinctive molecular structural configuration different from the changes observed in AD or any other tauopathy [311], there are nevertheless similarities in tauopathy between the two diseases [312]. In particular, in both diseases, the tau-related neuronal pathology is phosphorylated on the same amino acids, including tau phosphorylated on threonine 213; it shows immunoreactivity to both 3R-tau and 4R-tau; and all six tau isoforms are present [312,313,314]. Moreover, in general, studies on neurogenesis and migration show that following a head injury, there is an increase in neurogenesis and a redirection of the migration of new neuroblasts toward the site of the injury [216,315,316]. In the case of mild head trauma, the regions most affected and suffering the greatest damage due to rapid accelerations, decelerations, or rotational forces experienced by the brain appear to be primarily the perivascular regions and the depths of the cortical sulci [317]. Therefore, if the neurogenesis-migration system were to guide the distribution of tauopathy in CTE, we would expect to find the highest burden of tauopathy in the regions most stressed and damaged, because this is where the migration of new neurons carrying the seeds of tauopathy would be directed. Surprisingly, these sites around small cerebral vessels and deep within the cortical sulci coincide exactly with where tauopathy is concentrated in CTE [311,313,318,319,320,321].

### 4.10. Adult Neurogenesis and Early Anatomical Distribution of Tauopathy in AD: A Summary

In Table 3, I summarize all the predictions made by the theory of adult neurogenesis of AD regarding the early anatomical sites of tauopathy in AD. Figure 3 illustrates the close association between adult neurogenesis and the sites of early tauopathy in AD.

## 5. Conclusions

What appears to emerge from the literature is that there is a wealth of well-established anatomical data on the initial sites of tauopathy in AD; however, there seems to be a lack of equally robust and well-supported, widely accepted explanations for why these sites are affected. Furthermore, despite the same tau pathology, a relative heterogeneity appears to exist among the factors hypothesized as relevant for explaining its various localizations (e.g., selective cell vulnerability, connectivity, metabolic demand, genetic, age, etc.). The question I posed in this study is: “If tauopathy in AD begins in maturing neurons through adult neurogenesis, as hypothesized in the theory of adult neurogenesis of AD, where would we expect to find the first seeds of tauopathy and the first tangles?” Unexpectedly, this hypothesis makes a series of predictions regarding the different localizations of tauopathy in the early stages of AD that coincide with or are entirely consistent with the available empirical data regarding those same localizations. Not only that, but it provides a unified explanation for the heterogeneity of anatomical sites, which thus appears more parsimonious than the multiple explanatory factors proposed to date in the field. A third significant aspect of the theory is that it does not present itself as a new and alternative explanation tout court of the disease’s pathogenic mechanism compared to other explanations or theories, but rather seems to represent a missing piece of the puzzle. The neurogenesis-migration system appears, in fact, to integrate well with both the amyloid cascade theory [78,79] and the hypothesis regarding the primary role of tau in AD [322], with the theory of cell-to-cell transmission of tau [76,77,277], with other hypotheses concerning the initial deposition of Aβ (e.g., the antimicrobial theory) [323], and with the intervening role of glial cells [220]. Finally, the theory seems capable of explaining the localization of tauopathy in other tauopathies (e.g., CTE) and appears to be applicable to other proteinopathies as well (e.g., PDD, LATE, etc.). I believe that these aspects—predictive power, uniformity in explanation, integration with other theories, and generalizability to other diseases—make the model plausible and worthy of further investigation. If this model proves to be valid, it would open up new possibilities for treatment. The ability to influence adult neurogenesis would shift from being an adjunctive strategy to counteract and rehabilitate the symptoms of the disease to an intervention targeting the causes of the disease to halt the symptoms and/or prevent their onset.

## Figures and Tables

**Figure 1:**
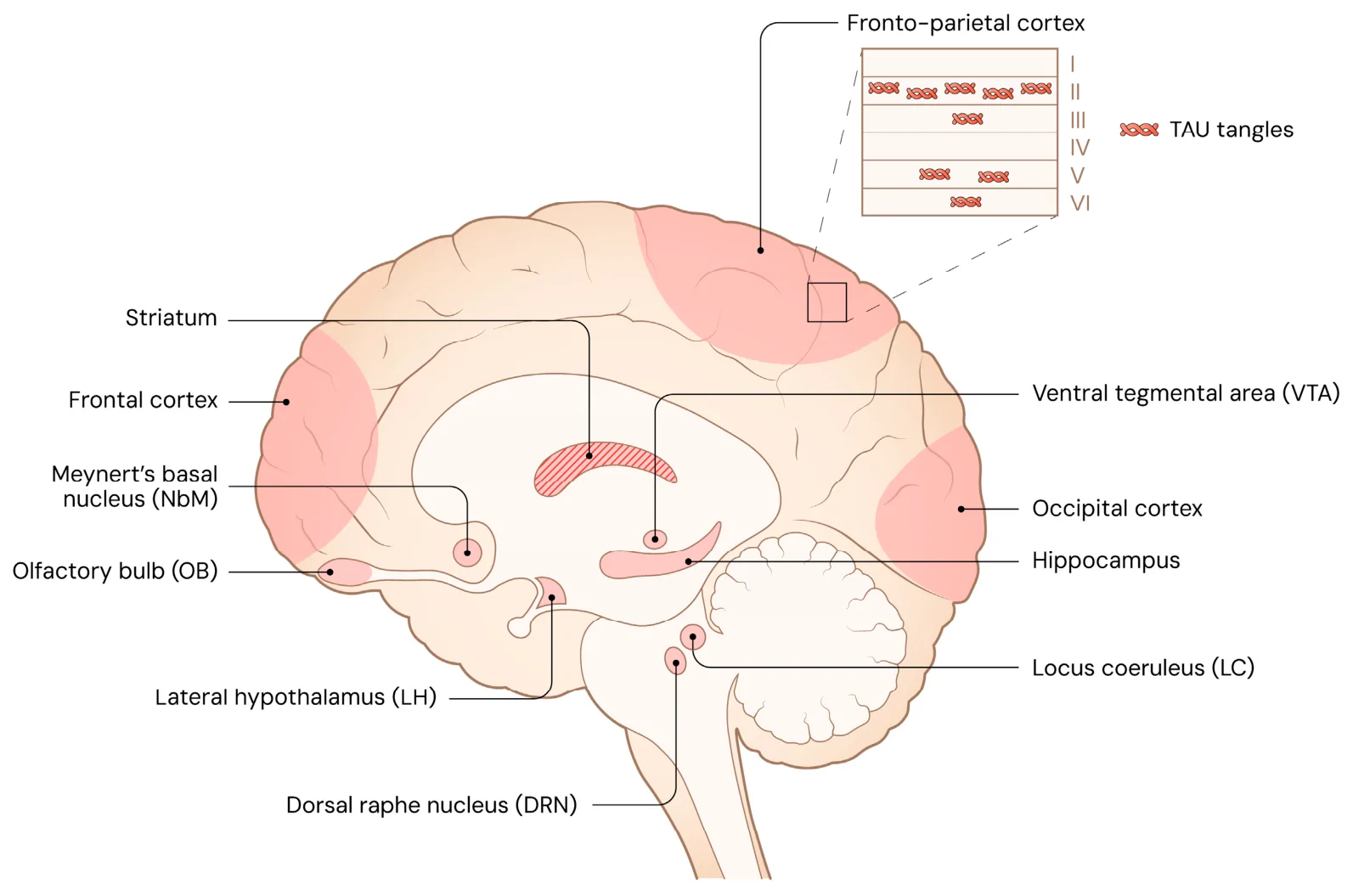
Sites of early tauopathy deposition in the AD brain. The figure shows the main cortical foci of early tauopathy development in AD. It depicts the mesial temporal focus, including the hippocampus and OB, as well as foci in the anterior (frontal cortex), dorsal (fronto-parietal cortex), and posterior (occipital cortex) regions, which correspond to the different clinical-radiological variants of AD (respectively, behavioral and disexecutive frontal syndromes, corticobasal syndrome, and PCA syndrome). The focus in the perisylvian and/or left temporo-parietal region corresponding to the logopenic variant has not been depicted for graphical reasons. The main subcortical sites of early-onset tauopathy (NbM, LH, VTA, DRN, LC) are also shown. The striatum has been colored differently because it does not represent a site of tauopathy onset like the others, but rather a site showing early and substantial accumulation of Aβ pathological deposits, especially in autosomal dominant AD (ADAD). The smaller inset at the top illustrates the tendency of tauopathy to more frequently affect cellular targets located in layer II of the cortex.

**Figure 2:**
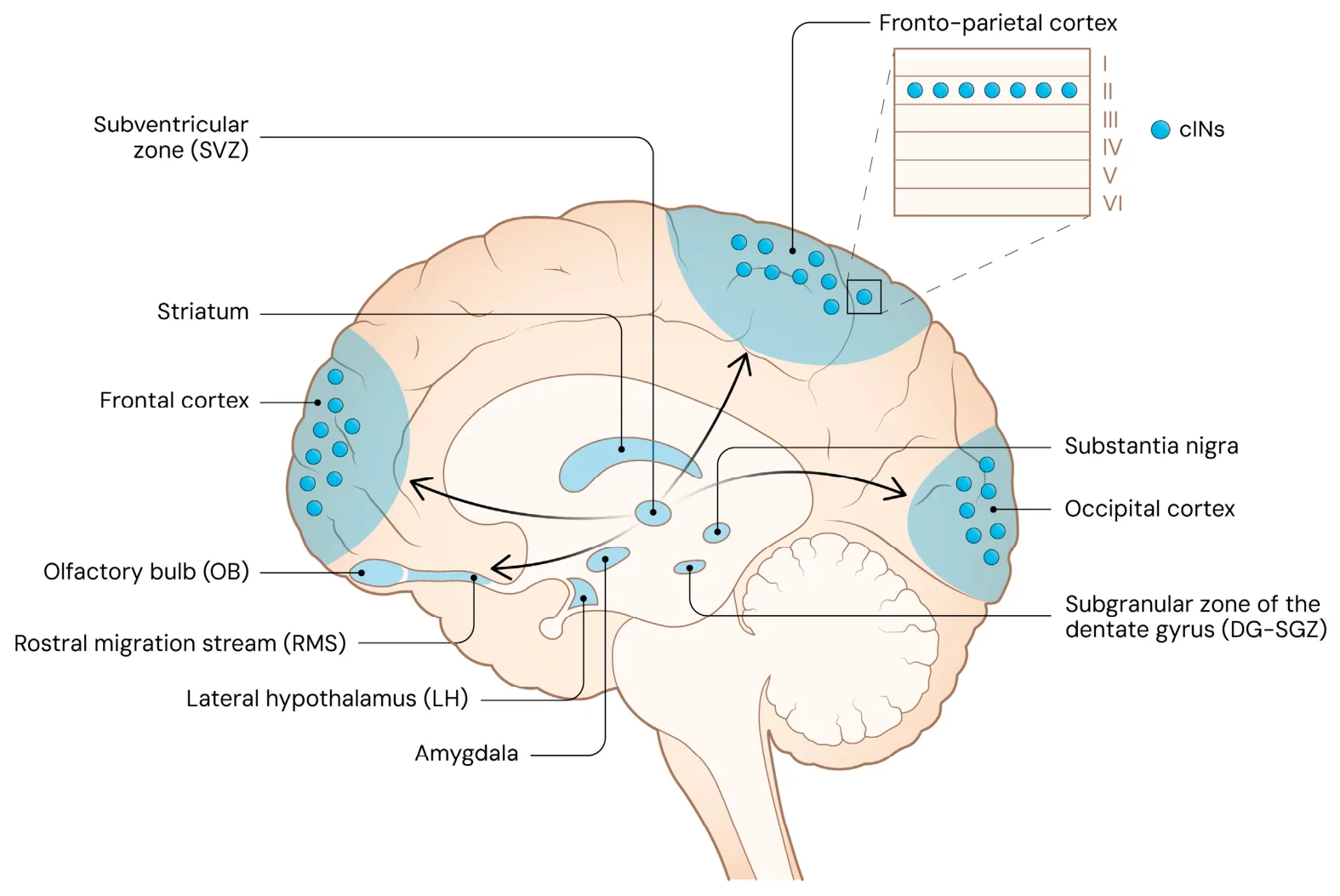
Sites of adult neurogenesis. The two main niches of adult neurogenesis are depicted: the subgranular zone in the dentate gyrus (DG-SGZ) and the subventricular zone (SVZ) along the lateral ventricles. Additionally, the cortical locations (anterior, dorsal, and posterior) of the pools of cortical immature neurons (cINs) from neurogenesis without division are shown. Furthermore, the possible neuronal migration pathways originating from the niche in the SVZ, along with the potentially reachable cortical areas, are depicted (black arrows), as well as the canonical migration pathway from the SVZ to the OB (rostral migration stream). Other non-canonical sites of adult neurogenesis in the striatum and amygdala are also shown. Finally, I have also included non-canonical sites of neurogenesis such as the lateral hypothalamus and substantia nigra, which have been documented primarily in other mammalian species. The small inset at the top illustrates the typical localization of pools of cortical immature neurons (cINs) within layer II of the cortex.

**Figure 3:**
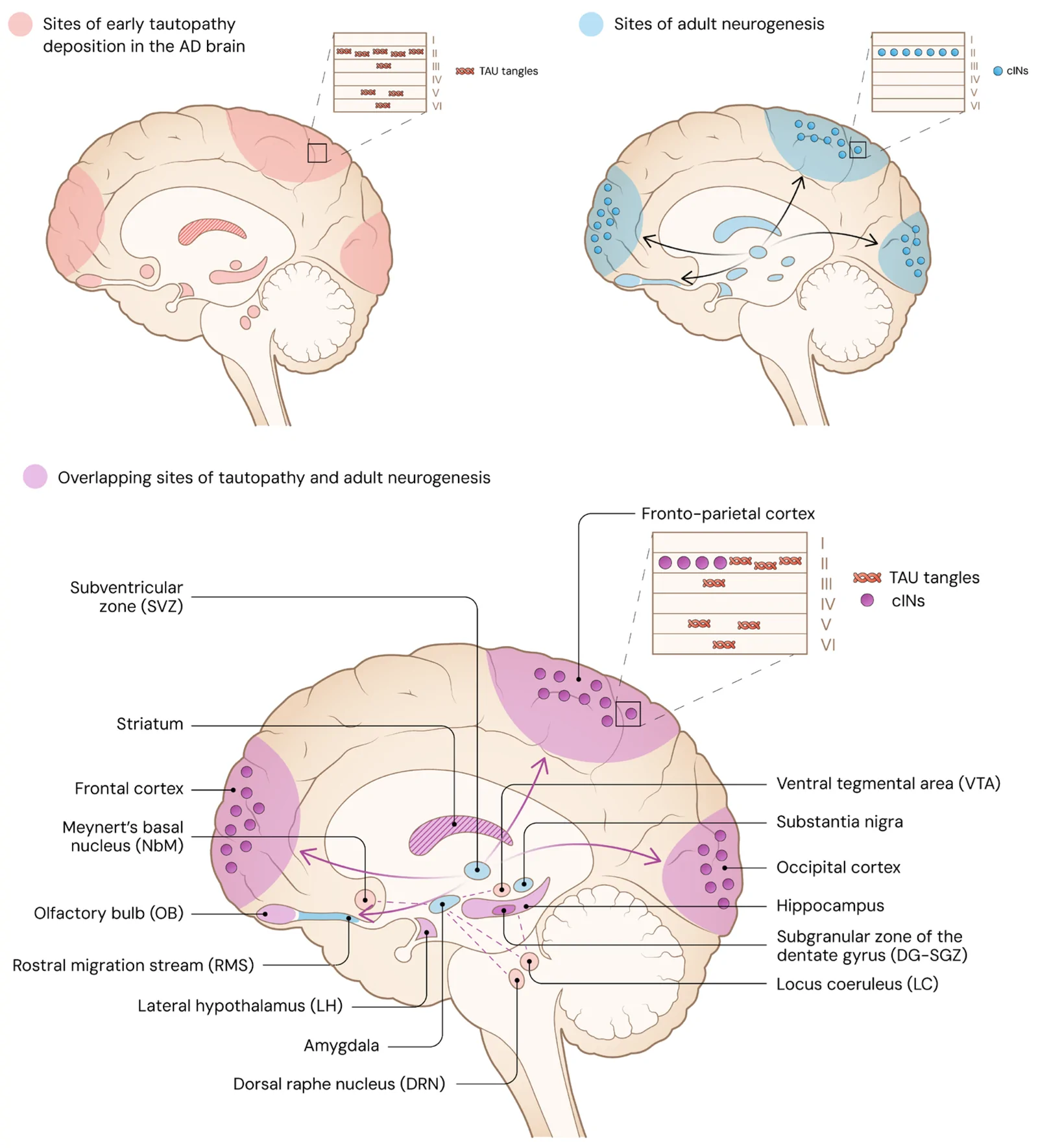
Overlapping sites of early tauopathy and adult neurogenesis. The figure shows the overlap (purple) between foci of the initial onset of tauopathy (pink) in AD and the brain sites involved in adult neurogenesis with and without cell division (blue). The location of the main niche for adult neurogenesis in the DG-SGZ of the hippocampus fully corresponds to the main focus of tauopathy onset in the middle temporal lobe. The OB is an area of early tauopathy onset and, at the same time, is the destination of the rostral migration stream originating from the SVZ niche. Furthermore, the possibility of long, non-canonical migrations toward the various regions of the anterior, dorsal, and posterior cortex starting from the SVZ neurogenesis niche (purple arrows), as well as the cortical localizations of pools of quiescent immature neurons (cINs), may overlap with the cortical foci of tauopathy onset characteristic of the clinical-radiological variants of AD. The lateral hypothalamus is a site of early tauopathy development in AD and, at the same time, is a non-canonical site of neurogenesis observed primarily in other mammalian species. The figure also illustrates the connections between subcortical nuclei where tauopathy appears early (NbM, VTA, DRN, LC) in AD and the amygdala, which has been identified as a non-canonical site of neurogenesis. Furthermore, the amygdala will also be affected by tauopathy at a relatively early stage (Braak stage III). The striatum is also depicted; unlike the other sites, it is a region of early and substantial deposition of Aβ pathology rather than tauopathy, and it has been recognized as a site of adult neurogenesis. Finally, the small panel at the top shows how the particular tendency of tauopathy to affect cellular targets—especially in layer II of the cortex—corresponds to the exclusive localization of pools of quiescent immature neurons in layer II.

**Table 1:** Early sites of tauopathy in AD and the main hypotheses proposed to explain vulnerability.

Anatomical Level and Further Related Issues	Initial Sites of Tauopathy in AD	Some of the Main Explanations
Intracellular compartments	Hyperphosphorylated tau, which precedes the formation of NFTs, accumulates primarily in the cell body and dendrites, with an initial preference for the distal portions of the dendrites [33,34].	According to the classical view in AD, hyperphosphorylated tau detaches from microtubules in the axon and then mislocalizes to the cell body and dendrites, where it ultimately forms neurofibrillary tangles [35,36].
Type of cells	Endogenous inclusions of pathological tau are found only in neurons. Pathological tau inclusions in glial cells may be present, but they are of external origin [37,38].	Astrocytes, oligodendrocytes, and microglia can internalize extracellular tau aggregates released by stressed or dying neurons, and in some cases, they fail to degrade tau, leading to secondary tau accumulation [39,40,41].
Cortical layer	Tauopathy in AD shows a predilection for affecting cellular targets in layer II of the cortex [45,50,51,58].	Neurons in layer II primarily contribute to corticocortical connectivity, especially when they interact with neurons in layer III [61]. The high synaptic load and activity in these hubs amplify the spread of tau protein within the inputs to the associative cortex, explaining why tauopathy first develops in these regions before spreading further [64].
Medial temporal lobe	Tauopathy in AD shows a particular predilection for affecting the middle temporal lobe (MTL), beginning in the TEC/EC complex [45,46,65,68,69,70].	(cell-autonomous factors) The first and most vulnerable neurons in AD are the excitatory neurons that express RORB and reelin in layer II of the EC [47,51,58]. Interneurons in layer II of the EC and in the superficial part of layer III are also vulnerable [50]. In particular, those that express parvalbumin (PV) are more vulnerable than those that express calbindin (CB), while those that express calretinin (CR) are the least vulnerable [50]. (non-cell-autonomous factors) Neurons in layer II primarily contribute to corticocortical connectivity. The high synaptic load and activity in these hubs amplify the spread of tau protein, explaining why tauopathy first develops in these regions before spreading further [64].
Subcortical nuclei	There is an early presence of tauopathy in subcortical nuclei (NbM, LC, VTA, LH, DRN) [99,100,101,102].	The reasons for the vulnerability of subcortical neurons to tau pathology are attributed primarily to their metabolic demands and to the oxidative stress resulting from neuromodulatory activity [105,106,107].
Cortex (phenotipic heterogeneity)	Four distinct patterns of tangle deposition and associated cortical damage are found in AD (hippocampal sparing, typical AD, limbic-predominant, and mild atrophy) [111]. Moreover, tauopathy and associated foci of degeneration are found in various regions of the cortex: posterior occipital-temporal and/or occipito-parietal regions (PCA syndrome); left temporo-parietal and/or perisylvian regions (logopenic variant); perirolandic regions (CBS-AD); anterior regions (frontal behavioral-executive variant) [126,127,128,129].	The research highlights several factors that contribute to phenotypic heterogeneity. Major drivers include genetic factors such as APOE ε4 alleles, sex, age, and polygenic risk scores, which modulate symptom onset and progression [113,146,147].
EOAD v/s LOAD	The distribution of tauopathy in EOAD is often different from that in LOAD. In general, EOAD primarily affects regions of the dorsal cortex, whereas LOAD affects the MTL [124,170].	Current research has identified genetic factors, interactions between tau and amyloid-β (Aβ), and the properties of brain networks as main factors that determine the different localization of tauopathy in EOAD compared to LOAD [175,176,177,178].
Hemispheric asymmetries	Studies consistently reveal asymmetric tau pathology accumulation in both typical and atypical AD [17].	The causes responsible for the asymmetric distribution of tauopathy in the cerebral hemispheres have not been fully elucidated. Some authors found that the degree of tau asymmetry was strongly associated with asymmetric amyloid-β (Aβ) patterns, not with differences in inter-hemispheric connectivity [182].
Clinical-anatomical convergence	The location of tauopathy and the corresponding regions affected by degeneration in a patient with AD may sometimes coincide exactly with those affected by another proteinopathy (e.g., lewy bodies pathology, 3R-tau, TDP-43, etc.) in another patient with a different disease (DLB, CBD, LATE, etc.) [183,184,185,186].	Misfolded proteins spread along anatomically connected pathways, so that disparate initial pathologies progressively involve the same networks on a large scale [187,188]. Certain highly connected cortical regions, or network hubs, are metabolically active and exposed to high levels of oxidative stress, making them selectively vulnerable in various diseases [189]. When different proteinopathies affect these nodes, the outcome is similar: network dysfunction and a predictable clinical syndrome [187,188].

Abbreviations: (NFTs): neurofibrillary tangles; (AD): Alzheimer’s disease; (MTL): medial temporal lobe; (RORB): RAR-related orphan receptor beta; (PV): parvalbumin; (CB): calbindin; (CR): calretinin; (NbM): cholinergic nucleus basalis of Meynert; (LC): noradrenergic locus coeruleus; (VTA): dopaminergic ventral tegmental area; (LH): orexinergic lateral hypothalamus; (DRN): serotonergic dorsal raphe nucleus; (APOE ɛ4): apolipoprotein epsilon 4; (PCA): posterior cortical atrophy; (CBS): corticobasal syndrome; (EOAD): early-onset Alzheimer’s disease; (LOAD): late-onset Alzheimer’s disease; (Aβ): beta-amyloid; (3R-tau): three microtubule-binding domain repeats tau; (TDP-43): Transactive Response DNA-binding Protein 43; (DLB): dementia with Lewy bodies; (CBD): corticobasal degeneration; (LATE): Limbic-predominant age-related TDP-43 encephalopathy.

**Table 2:** Factors that promote pathological hyperphosphorylation of tau protein in adult neurogenesis niches.

Variables	Postnatal Neurogenesis	Adult Neurogenesis	Description
Level of phosphorylation	High tau phosphorylation	High tau phosphorylation	Tau phosphorylation is developmentally regulated: it is higher in fetal neurons and decreases with age during development [190,191,192]. High tau phosphorylation is related to adult neurogenesis in both the V-SVZ and SGZ [193,194].
Tau Isoform	3R-tau isoform	3R-tau isoform	The tau isoform featured by the presence of three-repeat microtubule-binding domains (3R-tau) predominates at early developmental stages [195,196]. In newborn neurons in the niches of adult neurogenesis, 3R-tau is the primary isoform [193,197]. The 3R-tau isoform is said to facilitate PHFs, such as those seen in classical AD NFTs [198].
Sites of phosphorylation	Several tau phosphorylation sites associated with AD	Tau phosphorylation in fetal epitopes	Several sites of hyperphosphorylation of the fetal 3R-tau during development were the same as those in the AD brain [190,199,200,201]. High tau phosphorylation in fetal epitopes is related to adult neurogenesis in both the V-SVZ and SGZ [193,194], although fetal tau phosphorylation can be found in the adult brain in additional areas [192].
Tau kinase	Activated GSK-3β	Activated GSK-3β	GSK-3β is the first identified tau kinase [201,203] that plays a key role in AD-like tau hyperphosphorylation [204,205,206,207,208,209]. Interestingly, during development, expression of GSK-3β reaches its highest level in the late embryonic/early postnatal period, markedly decreasing with maturation into adulthood [210,211]. More importantly, activated GSK-3β is believed to be the primary tau kinase in newborn neurons during adult neurogenesis [193,212].
Migration	Easier migrations	Demanding migrations	New neurons require a high degree of plasticity to differentiate, project axons, integrate into the cell layer, and migrate [193,213]. Since phosphorylation reduces the affinity of the tau protein for microtubules [214], a high level of phosphorylation ensures a dynamic microtubule network [193,213] suited for the various functions involved in its development. In the large, evolutionarily developed brain of adult mammals, neuroblasts originating in the neurogenic niches must migrate long distances through a complex and generally inhibitory environment [215] made up of neuronal, glial, and vascular networks to reach their destination [216]. I speculate that demanding and long migration through the inhibitory environment of the adult brain could have the detrimental effect of further increasing tau hyperphosphorylation.
Clearance	Efficient autophagy	Inefficient autophagy	Autophagy may serve both a surveillance role by ensuring the quality of NSCs by degrading and eliminating intracellular components and aggregates and an elimination role by ensuring the removal of defective or damaged NSCs through its cell death promotion abilities [217,218]. Reduced autophagy during aging compared to the postnatal period may contribute to the accumulation of hyperphosphorylated tau aggregates in NSCs.

Abbreviations: (V-SVZ): ventricular–subventricular zone; (SGZ): subgranular zone; (3R-tau): three microtubule-binding domain repeats tau; (PHFs): paired helical filaments; (NFTs): neurofibrillary tangles; (GSK-3β): glycogen synthase kinase 3 beta; (NSCs): neural stem cells.

**Table 3:** A comparison of the initial sites of tauopathy and the predictions made by the theory of adult neurogenesis of AD.

Sites of Early Tauopathy in AD	Predictions Made by the Theory of Adult Neurogenesis of AD
Somatodendritic tauopathy	The idea that tauopathy begins to develop in immature neurons is entirely consistent with the somatodendritic localization of tauopathy in AD, because, in immature neurons—unlike in mature neurons—the tau protein is expressed at the somatodendritic level rather than the axonal level [222,223]. Not only is GSK-3β present at the somatodendritic level [231], but some studies have suggested the possibility that Aβ oligomers may also be present within the cell [219]. Thus, the key players in the amyloid theory (i.e., tau, Aβ and GSK-3β) could all be present in immature neurons. Furthermore, the absence of tauopathy in the axon would be consistent with this model, because the early presence of tauopathy seeds in the processes of the maturing neuron could conflict with the dynamics of axon specification [244,245]. Thus, the process that becomes the axon is likely the one with the least tauopathy. This model also appears more parsimonious than the classical view because there is no longer a need to postulate that the detachment of tau from axonal microtubules and its displacement toward the soma and dendrites underlie the pathogenesis of AD.
Endogenous tauopathy inclusions only in neurons	Although both neurons and glial cells, such as oligodendrocytes and astrocytes, are generated in niches during adult neurogenesis [255], the proposed theory is consistent with the fact that endogenous tauopathy in AD appears only in neurons. In fact, oligodendrocytes and astrocytes, which are capable of forming endogenous tau inclusions in other tauopathies, simply do not develop them in AD because they do not express the tau protein when immature [258,259,260].
Tauopathy in layer II	The preference of tauopathy in AD for cellular targets in layer II of the cortex shows a striking overlap with the site of division-free neurogenesis. Pools of immature cortical neurons in adults are in fact invariably found in layer II of the cortex [238,239,240,241,265,266,267].
Tauopathy in the EC/TEC and early spread in the MTL	The hippocampal localization of tauopathy in the most common classic amnestic form of AD fully corresponds to the site of the main niche of adult neurogenesis, which is located in the SGZ of the DG [269]. Not only that, but the high clearance activity present in the niche eliminates many new neurons carrying the first seeds of tauopathy and explains why, although tauopathy is thought to develop in immature neurons in the SGZ, tangles do not appear early in the DG and CA3 [270], which receive efferent projections from the DG. On the other hand, the new model of retrograde transmission from the DG to the EC predicts early involvement of the DG’s GABAergic interneurons, which have important connections with the SGZ and indeed show high vulnerability to tauopathy [221]. Furthermore, the model is consistent with data regarding early deterioration of the perforant path fibers between the DG and EC [92] and with the initial appearance of tauopathy in layer II of the EC, where input fibers from the DG arrive. Furthermore, the subsequent transmission of tauopathy from the EC to the TEC, occurring both anterograde and retrograde due to the reciprocal connections between the two areas, results in a massive accumulation of tauopathy seeds in the TEC compared to the EC, which instead receives the first tauopathy seeds only retrograde from the DG. This scenario thus provides the basis for a possible explanation of why the first tangles appear in the TEC. Not only that, but this model is consistent with the fact that the first tangles in TEC appear in layer II, precisely where the main input connections from EC arrive [83,84]. Finally, this scenario also explains why tangles appear in CA1 and the subiculum much earlier than in DG and CA3, despite there being no differences in vulnerability to tauopathy in the connecting fibers between EC and the first two regions compared to the latter two [48,94,95,96,97], and despite the fact that CA1 and the subiculum receive fibers originating from layer III of EC—which is not the first layer involved— while DG and CA3 receive connecting fibers originating from layer II of the EC, which is the first to be affected. In particular, the progression of tauopathy from the EC toward CA1 and the subiculum would be dictated by the fact that the transmission pathways between the EC and DG at this stage would already be impaired due to the prior retrograde passage of tauopathy seeds from the DG to the EC. Therefore, the only possible transmission pathways not yet deteriorated for tauopathy seeds would be exclusively those from the EC to CA1 and the subiculum. Finally, the OB, which is affected early by tauopathy in AD [66], is one of the main sites of adult neurogenesis, as it is the endpoint of the canonical migration pathway of RMS cells originating from the SVZ niche [269].
Early presence of tauopathy in subcortical nuclei	The discovery of neurogenesis niches in non-canonical areas, such as the hypothalamus, substantia nigra, and amygdala, in various species of mammals [282,283,284,285,286,287,288,289,290,291,292] provides the basis for a possible explanation of the early detection of tauopathy in subcortical nuclei. The lateral hypothalamus is, in fact, one of the regions involved early on by tauopathy in AD [100,101,102]. Furthermore, other nuclei involved early on, such as the locus ceruleus (LC), ventral tegmental area (VTA), nucleus basalis of Meynert (NbM), and dorsal raphe nucleus (DRN), have close connections with the amygdala and hypothalamus [99,100,101,102,103,104]. Even when considering neurogenesis without cell division, the available data support the significant presence of immature neurons in subcortical areas such as the amygdala and claustrum, the external capsule, and the corpus callosum [240,266].
Tauopathy in the cortex and phenotypic heterogeneity	The neurogenesis-migration model offers an explanatory framework highly compatible with the phenomenon of phenotypic heterogeneity in AD. The presence of a primary niche in the DG accounts for the typical and more frequent temporal-mesial involvement of the disease with its associated amnestic phenotype [269]. At the same time, the SVZ niche, together with non-canonical migration pathways toward cortical areas, or alternatively the cortical localizations of pools of quiescent immature neurons, account for the posterior, anterior, left perisylvian, and parietal epicenters of the disease’s phenotypic variants (i.e., PCA, behavioral-dysexecutive, logopenic, and corticobasal) [267]. Furthermore, the idea that multiple distinct sources of immature neurons are active simultaneously accounts for composite and less specific cognitive phenotypes, which are more common in clinical practice, such as multi-domain MCI [135]. Finally, by also considering possible neurogenic niches at the subcortical level, the model accounts for the heterogeneity of non-cognitive symptoms associated with the disease.
Localization of tauopathy in EOAD v/s LOAD	The progressive and uneven loss of efficiency and functionality of the different neurogenic niches during aging, with a recognized earlier deactivation of the SVZ compared to the SGZ in DG [297,298,299], and at the same time of pools of quiescent neurons in cortical areas compared to those at the temporal level [267], explains the different localization of tauopathy between EOAD, which occurs mainly at the cortical level, and LOAD, which manifests mainly at the hippocampal level [124,170].
Hemispheric asymmetries in tauopathy	The presence of two homologous systems of neurogenesis-migration and pools of immature neurons in the two cerebral hemispheres provides the basis for explaining hemispheric asymmetries in the localization or extent of tauopathy in AD [17], as well as the selective involvement of regions in a single hemisphere in certain phenotypic variants (e.g., logopenic, right PCA, etc.).
Clinical-anatomical convergence	Finally, in the new theory, the neurogenesis-migration system that determines where tauopathy develops and where it is initially distributed in the brain, is relatively independent of the pathogenic mechanism that causes tauopathy. This relative independence allows us to hypothesize that the same neurogenesis-migration system may also be involved in other proteinopathies and may guide their anatomical distribution in the brain. This fact lays the groundwork for explaining the clinical-anatomical convergence observed in neurodegenerative diseases. Moreover, surprisingly, the same neurogenesis-migration system appears capable of explaining the distinctive localization of tauopathy in a disease other than AD, such as CTE [311,313,317,318,319,320,321].

Abbreviations: (AD): Alzheimer’s disease; (GSK-3β): glycogen synthase kinase 3 beta; (Aβ): beta-amyloid; (SGZ): subgranular zone; (DG): dentate gyrus; (CA3): cornu ammonis 3; (EC): entorhinal cortex; (GABA): gamma-aminobutyric acid; (TEC): transentorhinal cortex; (CA1): cornu ammonis 1; (OB): olfactory bulb; (RMS): rostral migratory stream; (SVZ): subventricular zone; (LC): locus coeruleus; (VTA): ventral tegmental area; (NbM): nucleus basalis of Meynert; (DRN) dorsal raphe nucleus; (PCA): posterior cortical atrophy; (MCI): mild cognitive impairment; (EOAD): early-onset Alzheimer’s disease; (LOAD): late-onset Alzheimer’s disease; (CTE): chronic traumatic encephalopathy.

## Data Availability

No new data were created or analyzed in this study. Data sharing is not applicable to this article.
